# Pharmacotherapy for mood and anxiety disorders in older people with intellectual disability in comparison with the general population

**DOI:** 10.1186/s12888-019-2191-7

**Published:** 2019-08-01

**Authors:** Anna Axmon, Nadia El Mrayyan, Jonas Eberhard, Gerd Ahlström

**Affiliations:** 1 EPI@LUND, Division of Occupational and Environmental Medicine, Department of Laboratory Medicine, Lund University, SE-221 00 Lund, Sweden; 2 Department of Health Sciences, Faculty of Medicine, Lund University, SE-221 00 Lund, Sweden; 3 Division of Psychiatry, Department of Clinical Sciences Lund, Lund University, SE-25187 Helsingborg, Sweden

**Keywords:** Aging, Down syndrome, Drug prescription, Mental retardation, Sedatives

## Abstract

**Background:**

People with intellectual disability (ID) have high prevalence of psychiatric disorders, but even higher rates of prescription of psychotropic drugs.

**Methods:**

Using Swedish national registers, we identified a group of older people with ID and diagnosis of mood disorders (ICD-10 codes F32-F39) and/or anxiety (ICD-10 code F4) during 2006–2012 (*n* = 587) and a referent group of people from the general population with the same diagnoses during the same time period (*n* = 434). For both groups, we collected information on prescription of anxiolytics, hypnotics and sedatives, antidepressants, and GABA-agonists.

**Results:**

Among those with a diagnosis of anxiety, people with ID were more likely than those in the general population to be prescribed anxiolytics (Relative Risk 1.32 [95% Confidence Interval 1.19–1.46]) and GABA-agonists (1.10 [1.08–1.31]). Moreover, among those with anxiety but without mood disorders, ID was associated with increased prescription of antidepressants (1.20 [1.03–1.39]). Within the ID cohort, behaviour impairment and MSP (i.e. moderate, severe, or profound) ID was associated with increased prescription of anxiolytics, both among those with anxiety (1.15 [1.03–1.30] for behaviour impairment and 1.23 [1.10–1.38] for MSP ID) and among those with mood disorders (1.14 [0.97–1.35] for behaviour impairment and 1.26 [1.04–1.52] for MSP ID). Moreover, MSP ID was associated with increased prescription of GABA-agonists among those with anxiety (1.23 [1.10–1.38]).

**Conclusions:**

The excess prescription of anxiolytics but not antidepressants may suggest shortages in the psychiatric health care of older people with intellectual disability and mood and anxiety disorders.

## Background

People with intellectual disability (ID) have a high risk of psychiatric diagnoses, such as depression mood disorders, and anxiety [1–3]. Indeed, psychiatric diagnoses is more common among people with ID than in the general population [4]. This even if diagnosing psychiatric disorders in people with ID, and especially those with severe ID, may be complicated by symptoms being masked by behavioural impairment [5]. Moreover, diagnosing psychiatric disorders in people with ID is difficult due to their decrease of communication abilities and thereby the ability to properly describe symptoms [6].

Over the recent decades, the life expectancy among people with ID have been increasing [7]. As a consequence, they now live longer with psychiatric comorbidities, and are also at higher risk of age-related psychiatric disorders, such as dementia. This, in turn, increases their risk of prescription of psychotropic drugs. As long as the increase in prescription reflects the increase in disorders, there should be no cause for concern. However, there are suggestions that this might not be the case [8]. Antipsychotic drugs are often prescribed off-label to manage behavioural impairment [8–10]. This in spite the lack of evidence of their effectiveness for this [9], and the increased risk of side effects found for them in people with ID [11, 12] which may result in a decreased quality of life [13].

We have previously reported that, in comparison with their age-peers in the general population, older people with ID receive different treatment for pain [14], diabetes [15], hypertension [15], and asthma and chronic respiratory disorders [16]. This, taken together with the high rates of prescription of psychotropics among older people with ID, raises the question of whether people with ID and psychiatric disorders receive the same pharmaceutical treatment as people in the general population with the same disorders. Therefore, the aim of this study was to investigate, given a diagnosis of mood disorders or anxiety, if there are any differences between older people with ID and their age peers in the general population with respect to prescription of antidepressants, anxiolytics, hypnotics and sedatives, or GABA-agonists. Moreover, to study potential effects of behavioural impairment, severity of ID, sex, and year of birth on prescription of these drugs among older people with ID and diagnosis of mood disorders or anxiety.

## Methods

### Study design

The present study is a register study, using register both to define the study cohorts and to collect data on subgroups and outcomes. Based on the availability of outcome data (drug prescription), as explained below, the study period is 2006–2012. However, when identifying subgroups based on severity of ID and presence of behaviour impairment, data from 2002 to 2012 are used.

### Registers

According to Swedish law, all people with ID and/or autism spectrum disorder (ASD) can apply for service and support for their daily living from the municipality. All service and support provided under this law is reported to the Swedish National Board of Health and Welfare, and recorded in the so-called LSS register. The register contains information on which services have been provided and by which municipality, but not regarding specific diagnoses of the ID and/or the ASD disabilities.

The Swedish National Patient Register contains information on visits made to inpatient and outpatient specialist health care in Sweden. For each visit, one primary and up to 21 secondary diagnoses are recorded according to the International Statistical Classification of Diseases and Related Health Problems 10th Revision (ICD-10). The primary diagnosis is supposed to reflect the cause of the visit, as determined by the end of the visit. As secondary diagnoses, conditions that are of importance in the treatment or diagnosing of the cause of the visit should be recorded.

All dispensed prescribed drugs in Sweden are recorded in the Drug Prescription Register, regardless if the prescription was made in primary or specialist health care, or in a public or private health care setting. Drugs are recorded according to the Anatomical Therapeutic Chemical (ATC) classification system, in which the active substances are classified in a hierarchy with five different levels. The register was established in July 2005, making 2006 the first year with complete available data.

### Study cohorts

**Fig. 1:**
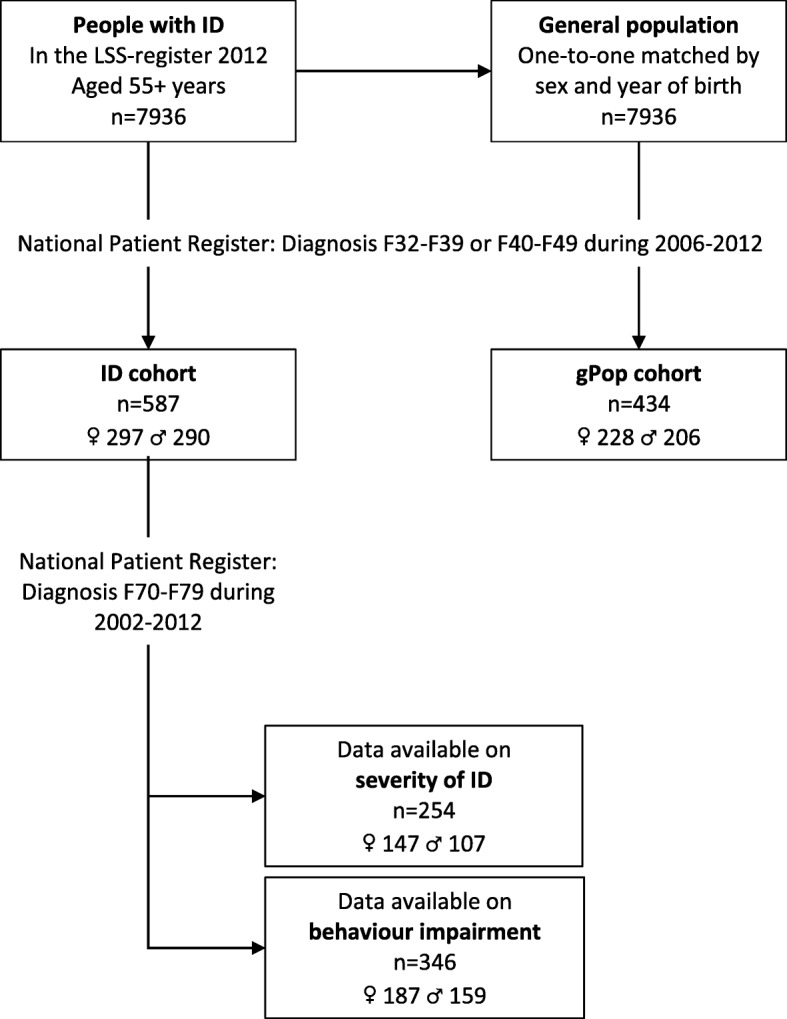
Flow chart of study cohort and subgroup identification

The process of establishing of study cohorts and subgroups is displayed in Fig. 1. In a first step, we identified 7936 people with at least one measure of support for people with ID and/or ASD during 2012, aged at least 55 years and alive at the end of 2012. Thus, we used having support intended for people with ID and/or ASD as a proxy for having ID. Statistics Sweden provided us with references from the general population, one-to-one matched to the ID cohort by sex and year of birth. People who had received LSS support during 2012 were not allowed to be included in the reference cohort. However, no restrictions were made with respect to having received LSS support previous years, or having a diagnosis of ID during or before the study period.

In the next step, we identified people with at least one diagnosis of mood disorders (F32-F39 in ICD-10) or anxiety (F40-F49). The 587 people (290 men and 297 women) from the LSS register with at least one such diagnosis comprised the ID cohort, and the 434 people (206 men and 228 women) from the general population the gPop cohort.

Thirdly, within the ID cohort, we identified people with at least one diagnosis of ID (F7 in ICD-10) during 2002–2012 (i.e. from 5 years before the start of the study period). These were categorized as having mild (F70; *n* = 153), MSP (moderate/severe/profound; F71/F72/F73; *n* = 101) or other/unspecified ID (F78/F59; *n* = 92). Moreover, among those with a diagnosis of ID, we used the fourth digit of the ICD-10 code to classify each individual as either having (F7X.1 [significant impairment of behaviour requiring attention or treatment] and F7X.8 [other impairments of behaviour]; *n* = 147) or not having (F7X.0 [with the statement of no, or minimal, impairment of behaviour] and F7X.9 [without mention of impairment of behaviour]; *n* = 199) behaviour impairment. In the analyses of severity of ID, people with other/unspecified ID were excluded.

### Drugs

Using the Drug Prescription Register, we identified all records of prescriptions of anxiolytics (ATC-code N05B), hypnotics and sedatives (N05C; excluding melatonin), and antidepressants (N06A) for those with at least one diagnosis of mood disorders or anxiety. In all analyses, the drugs were aggregated into the four-level ATC code, i.e. N06A, N05B, and N05C. We also investigated drugs with strong anxiolytic effects via the GABA (Gamma-Aminobutyric Acid)-system, i.e. GABA-agonists. In this group, we included benzodiazepine derivatives (N05BA and N05CD), benzodiazepine related drugs (N05CF), aldehydes and derivatives (N05CC), and clomethiazole (N05CM02). The individual drugs included in these four groups are described in Table 1.

**Table 1:** Drugs included in the four investigated pharmaceutical groups, and number of people in a cohort of people with intellectual disability (ID) and a reference group from the general population (gPop) with at least one prescription during the study period

	gPop	ID
Anxiolytics		
N05BA01 diazepam^a^	83	198
N05BA02 chlordiazepoxide^a^	< 5	< 5
N05BA04 oxazepam^a^	157	264
N05BA06 lorazepam^a^	< 5	19
N05BA09 clobazam^a^	< 5	< 5
N05BA12 alprazolam^a^	42	58
N05BB01 hydroxyzine	128	162
N05 BC01 meprobamate	< 5	< 5
N05BE01 buspirone	15	22
Hypnotics and sedatives		
N05CC01 chloral hydrate^a^	< 5	< 5
N05CD02 nitrazepam^a^	26	43
N05CD03 flunitrazepam^a^	16	14
N05CD05 triazolam^a^	< 5	< 5
N05CD08 midazolam^a^	< 5	< 5
N05CF01 zopiclone^a^	173	214
N05CF02 zolpidem^a^	112	83
N05CF03 zaleplon^a^	8	< 5
N05CM02 clomethiazole^a^	< 5	57
N05CM05 scopolamine	< 5	< 5
N05CM06 propiomazine	164	186
N05CM09 Valerianae radix	< 5	< 5
Antidepressants		
N06AA04 clomipramine	14	42
N06AA06 trimipramine	< 5	< 5
N06AA09 amitriptyline	43	34
N06AA10 nortriptyline	5	< 5
N06AA21 maprotiline	< 5	< 5
N06AB03 fluoxetine	31	29
N06AB04 citalopram	124	206
N06AB05 paroxetine	19	33
N06AB06 sertraline	100	127
N06AB08 fluvoxamine	< 5	< 5
N06AB10 escitalopram	73	88
N06AF03 phenelzine	< 5	< 5
N06AF04 tranylcypromine	< 5	< 5
N06AG02 moclobemide	< 5	< 5
N06AX02 tryptophan	< 5	< 5
N06AX03 mianserin	25	27
N06AX11 mirtazapine	148	141
N06AX12 bupropion	27	16
N06AX16 venlafaxine	78	54
N06AX18 reboxetine	9	< 5
N06AX21 duloxetine	59	33
N06AX22 agomelatine	9	< 5

^a^Also included in the group “GABA-agonists”

### Statistics

Analyses were performed on data aggregated to the entire study period, i.e. using yes/no variables describing if each person had at least one diagnosis/prescription during the study period. Diagnoses were assessed in five different ways: 1) having at least one diagnosis of mood disorders, regardless of whether anxiety was a co-morbidity (**mood disorders**), 2) having at least one diagnosis of anxiety, regardless of whether mood disorders was a co-morbidity (**anxiety**), 3) having at least one diagnosis of mood disorders but no diagnosis of anxiety (**mood disorders only**), 4) having at least one diagnosis of anxiety but no diagnosis of mood disorders (**anxiety only**), and 5) having at least one diagnosis of mood disorders and at least one diagnosis of anxiety (**both diagnoses**).

Relative risks (RRs) with 95% confidence intervals (CIs) were estimated using generalized linear models (GLMs). This was done in order to compare the ID and the gPop cohort, but also to analyse potential effects of severity of ID, presence of behaviour impairment, sex, and year of birth (dichotomized at the median date of birth) within the ID cohort. All analyses were performed in IBM SPSS Statistics 25.0. Two-sided *p*-values lower than 0.05 were considered statistically significant.

## Results

In the ID cohort, 31 people (5%) were not prescribed any of the investigated drugs during the study period, compared to 43 (10%; RR 0.53, 95% CI 0.34–0.83) in the gPop cohort. Moreover, 256 (44%) in the ID cohort and 178 (41%; RR 1.06, 95% CI 0.92–1.23) in the gPop cohort were prescribed drugs within all four groups investigated (anxiolytics, hypnotics and sedative, antidepressants, and GABA-agonists).

Among those with anxiety, people with ID were more likely to be prescribed anxiolytics and GABA-agonists, regardless of whether a diagnosis of mood disorders was present (Tables 2 and 3). Among those with anxiety but without mood disorders, people with ID were more likely to be prescribed antidepressants. The results were similar when adjusting for sex and year of birth (data not shown).

**Table 2:** Number of people with at least one diagnosis of mood disorders (F32-F39 in ICD-10) or anxiety (F4) during the study period in a group of people with intellectual disability (ID) and a referent group from the general population (gPop), as well as stratified on presence of behaviour impairment, severity of ID, sex, and date of birth (before/after median)

	Total	Mood disorders^a^		Anxiety^a^		Mood disorders only		Anxiety only		Both diagnoses	
		n	%	n	%	n	%	n	%	n	%
ID	587	336	57	354	60	233	40	251	43	103	18
gPop	434	264	61	276	64	158	36	170	39	106	24
Within the ID cohort											
Without behaviour impairment	147	116	79	117	80	82	56	83	56	34	23
With behaviour impairment	199	85	43	85	43	62	31	62	31	23	12
Mild ID	101	89	88	97	96	56	55	64	63	33	33
Moderate/severe/profound ID	153	60	39	60	39	41	27	41	27	19	12
Women	290	181	62	167	58	130	45	116	40	51	18
Men	297	155	52	187	63	103	35	135	45	52	18
Born before median date of birth	294	176	60	161	55	133	45	118	40	43	15
Born after median date of birth	293	160	55	193	66	100	34	133	45	60	20

^a^Any diagnosis of mood disorders/anxiety regardless of whether a diagnosis of the other investigated disorder is present

**Table 3:** Relative risks (RRs) with 95% confidence intervals (CIs) for prescription among people with intellectual disability (ID) compared with a referent cohort from the general population (gPop) and for demographic factors within the ID cohort, stratified by diagnoses of mood disorders (F32-F39 in ICD-10) and/or anxiety (F4). Bold indicates statistically significant differences in prescription

			Anxiolytics	Hypnotics and sedatives	Antidepressants	GABA-agonists
Cohort comparisons	ID (n = 587) vs gPop (n = 434)	Mood disorders^a^	1.03 (0.92–1.15)	0.93 (0.83–1.04)	0.98 (0.93–1.03)	1.01 (0.92–1.10)
		Anxiety^a^	**1.32 (1.19–1.46)**	1.04 (0.91–1.17)	1.07 (0.98–1.17)	**1.19 (1.08–1.31)**
		Mood disorders only	1.02 (0.88–1.20)	0.86 (0.73–1.01)	0.97 (0.90–1.03)	0.98 (0.87–1.12)
		Anxiety only	**1.54 (1.31–1.80)**	1.07 (0.89–1.28)	**1.20 (1.03–1.39)**	**1.31 (1.13–1.51)**
		Both diagnoses	1.10 (0.97–1.25)	1.09 (0.95–1.25)	1.01 (0.95–1.08)	1.10 (0.99–1.22)
Within the ID cohort	With (n = 147) vs without (n = 199) behaviour impairment	Mood disorders^a^	1.14 (0.97–1.35)	1.12 (0.91–1.37)	0.95 (0.85–1.05)	1.07 (0.93–1.24)
		Anxiety^a^	**1.15 (1.03–1.30)**	1.07 (0.86–1.32)	1.01 (0.88–1.16)	1.09 (0.97–1.22)
		Mood disorders only	1.10 (0.89–1.37)	1.23 (0.93–1.62)	0.94 (0.81–1.08)	1.12 (0.93–1.35)
		Anxiety only	1.12 (0.97–1.28)	1.15 (0.86–1.54)	1.04 (0.86–1.25)	1.13 (0.97–1.31)
		Both diagnoses	**1.25 (1.02–1.54)**	0.95 (0.73–1.24)	0.97 (0.83–1.13)	1.00 (0.85–1.18)
	Moderate/severe/profound (n = 101) vs mild (n = 153) ID	Mood disorders^a^	**1.26 (1.04–1.52)**	1.03 (0.81–1.32)	0.92 (0.80–1.06)	1.15 (0.97–1.35)
		Anxiety^a^	**1.23 (1.10–1.38)**	1.03 (0.81–1.32)	0.99 (0.85–1.15)	**1.23 (1.10–1.38)**
		Mood disorders only	1.25 (0.95–1.64)	1.25 (0.88–1.82)	0.91 (0.75–1.11)	1.25 (0.98–1.60)
		Anxiety only	**1.19 (1.04–1.38)**	1.22 (0.86–1.72)	1.02 (0.83–1.26)	**1.36 (1.16–1.59)**
		Both diagnoses	**1.32 (1.09–1.60)**	0.84 (0.62–1.13)	0.95 (0.80–1.14)	1.04 (0.90–1.21)
	Women (*n* = 297) vs men (*n* = 290) vs	Mood disorders^a^	1.06 (0.92–1.22)	1.00 (0.86–1.17)	1.01 (0.94–1.09)	1.00 (0.89–1.13)
		Anxiety^a^	1.04 (0.94–1.15)	0.91 (0.78–1.07)	1.05 (0.94–1.17)	0.99 (0.89–1.10)
		Mood disorders only	1.10 (0.90–1.34)	0.91 (0.73–1.14)	1.01 (0.92–1.12)	0.99 (0.84–1.17)
		Anxiety only	1.03 (0.91–1.17)	**0.73 (0.58–0.93)**	1.05 (0.89–1.24)	0.95 (0.82–1.10)
		Both diagnoses	1.04 (0.90–1.22)	**1.23 (1.03–1.47)**	1.02 (0.93–1.11)	1.06 (0.94–1.20)
	Before (*n* = 294) vs after (*n* = 293) median date of birth	Mood disorders^a^	1.02 (0.89–1.17)	1.15 (0.98–1.35)	1.06 (0.99–1.14)	0.96 (0.85–1.08)
		Anxiety^a^	1.04 (0.94–1.14)	1.17 (0.99–1.38)	**1.16 (1.03–1.31)**	1.03 (0.92–1.14)
		Mood disorders only	0.91 (0.74–1.11)	1.09 (0.87–1.37)	1.04 (0.95–1.14)	0.87 (0.73–1.04)
		Anxiety only	1.00 (0.88–1.13)	1.17 (0.93–1.48)	**1.18 (1.00–1.40)**	1.02 (0.88–1.17)
		Both diagnoses	1.11 (0.94–1.31)	1.10 (0.91–1.32)	1.08 (0.98–1.20)	1.01 (0.89–1.14)

^a^Any diagnosis of mood disorders/anxiety regardless of whether a diagnosis of the other investigated disorder is present

Within the ID cohort, people with behaviour impairment and anxiety were more likely than those with anxiety but without behaviour impairment to be prescribed anxiolytics (Tables 2 and 3). No differences were found between those with and without behaviour impairment with respect to prescription of any of the other investigated drug groups. Among those with at least one diagnosis of mood disorders, MSP (moderate/severe/profound) ID was associated with increased prescription of anxiolytics. However, when excluding those with diagnosis also of anxiety, the increase was no longer statistically significant. MSP ID was also associated with prescription of both anxiolytics and GABA-agonists among those with a diagnosis of anxiety. Adjusting for sex and year of birth did not change the effect estimates more than marginally (data not shown).

Compared to women with ID and diagnosis of mood disorders or anxiety, men were less likely to be prescribed hypnotics and sedatives when diagnosed with a combination of mood disorders and anxiety (Tables 2 and 3). They were also less likely to be prescribed antidepressants when diagnosed with anxiety. Also among those with diagnosis of both mood disorders and anxiety, men were less likely than women to be prescribed GABA-agonists. Among those with diagnosis of anxiety, being born after the median date of birth (October 31, 1951) were more likely to be prescribed hypnotics and sedatives. No other effects of year of birth were found.

## Discussion

Among older people with anxiety, we found excess prescriptions of anxiolytics, antidepressants, and GABA-agonists among people with ID in comparison with the general population. Also, behaviour impairment and MSP (i.e. moderate, severe, or profound) ID were associated with higher prescription of anxiolytics among those with anxiety.

The present study has several methodological strengths, the major one being the use of national registers to identify the study groups and the outcome (i.e. drug prescription). Drawbacks, however, are that data were left truncated (i.e. diagnoses and prescriptions may be present before the start of the study period but unknown to us) and that we had no information regarding possible diagnoses made in primary health care. Thus, we can never with certainty claim that a diagnosis came before a prescription, or vice versa. Therefore, we chose to perform all analyses on data aggregated to the entire study period, i.e. using yes/no variables describing if each person had at least one diagnosis/prescription during the study period.

A potential weakness with the study could be the use of ICD-codes rather than clinical diagnoses to identify psychiatric disorders. However, the diagnoses from the Swedish National Patient Register are the best proxies of clinical diagnoses available at a national level. Moreover, as our focus was on drug treatment and not prevalence of psychiatric disorders, misclassification of disorders due to the use of ICD-10 codes should not have had a major impact on the results of the study.

Another potential weakness is the use of the LSS register to identify people with ID. This will potentially have caused failure to include people with ID if they do not receive any help to manage their daily lives. The Swedish social support system is designed so that people with need of assistance should receive this from the state, county, or municipality. It does not depend on family members or friends acting as informal care givers. Thus, a majority of people with ID receive support according to the LSS law, with higher numbers in older populations as they need more assistance and are less likely to have parents still alive and in condition to care for them. Thus, considering the age group studied, we believe that we have included a large fraction – if not all – people with ID in Sweden.

We have previously found that older people with ID are more likely than their age-peers in the general population to be prescribed anxiolytics [11] and the GABA-agonist benzodiazepine [17]. At a first glance, this may be interpreted as a result of higher occurrence of psychiatric diagnoses in this group [4]. However, in the present study we investigated prescription of these drugs among those with a diagnosis of anxiety. Thus, differences between people with ID and the general population regarding the prevalence of anxiety cannot explain the discrepancies in prescriptions, as all people in the analyses had such a diagnosis. Even so, although the prevalence does not come into play here, we cannot rule out differences in severity or duration of the anxiety symptoms. If people with ID tend to have anxiety of greater severity or for longer periods of time, this could potentially explain the increased prescription associated with diagnosis of anxiety. Moreover, the national patient register comprises only visits to inpatient and outpatient specialist health care, and not primary health care. Thus, we have failed to include people with diagnosis of anxiety in primary health care only, i.e. those that may be suspected to be less severe cases. If selection of people from primary health care into specialist health care is such that referred patients with ID have more severe anxiety than referred patients from the general population, this could partly explain the differences in prescription. However, previous studies have reported that people with ID are less, not more, likely to be referred to specialist health care [18]. Instead, the increased prescription of anxiolytics among those with behaviour impairment and both anxiolytics and GABA-agonists among those with MSP ID suggest that it is not the anxiety per se that causes the discrepancy between people with ID and the general population, but rather a behaviour that may be considered difficult and problematic for caregivers. It is the clinical experience of one of the authors (JE) that carers at assisted housing for people with ID often make explicit requests for prescription of sedatives drugs when accompanying a person with challenging behaviour. When faced with this in combination with administrative shortages, such as having insufficient staff and time, psychiatrists may be likely to comply with the carer and prescribe e.g. anxiolytics rather than consider alternate treatments, such as antidepressants. A further aspect is the potential difficulties in supplying psychotherapy and social treatment as a complement to pharmacological treatment among people with ID. Previous studies have found that, in people with ID other treatment modalities than medication, there is a lack of availability of resources and accessibility of facilities which in turn may lead to excess prescription of medication [19, 20].

Contrary to the results regarding anxiety and anxiolytics, no excess prescription of antidepressants was found for people with ID among those with mood disorders, nor did behaviour impairment or MSP ID carry any increased risk of prescription. Continuing the reasoning above, this could indicate that mood disorders occur with similar severity and duration among people with ID as in the general population. Another interpretation could be that mood disorders are associated with difficult behaviour to a lesser extent than anxiety among people with ID. Previous studies regarding challenging behaviour associated with depression in people with ID are inconclusive. Whereas some find aggression and screaming among people with ID and depression, especially for those with severe and profound ID [5, 21], other note no close association between self-injury or aggression and depression [22]. Future research is needed to understand the underlying mechanisms regarding depression, challenging behaviour, and drug prescription among people with ID.

In summation, that prescription of psychotropics is common among people with ID has been reported before, both for the cohorts included in the present study [11, 17] and in other populations [23]. The present study shows that this excess is present even when restricting the study groups to those with diagnosis of anxiety and mood disorders, and that the underlying reason for prescription must be sought elsewhere than in the prevalence of these diagnoses. It also provide further evidence that difficult behaviour may be one such underlying reason, something that has been suggested previously [8, 24]. This may be problematic, as there is insufficient evidence to support the use of psychotropics for challenging behaviour [25, 26], and there is a high risk of adverse events and negative influence on the quality of life associated with psychotropic drugs among people with ID [13].

## Conclusions

The excess prescription of anxiolytics but not antidepressants may suggest shortages in the psychiatric health care of older people with intellectual disability and mood and anxiety disorders. Health care provision needs to be adapted to ensure this particularly vulnerable group receives proper health care. This may, for example, be done by increasing the quantity of and accessibility to psychiatric health care. Moreover, psychiatrist need to consider antidepressants as an option in treating psychiatric disorders rather than routinely prescribe drugs with sedative effects. More research in this field is needed. Also, health care provision needs to be adapted to ensure that this particularly vulnerable group receives proper health care in conjunction with other therapies intended to improve their quality of life.

## Data Availability

The data in the present study contains sensitive information on a very vulnerable group, i.e. people with ID. Even though the data are anonymized, it contains enough details to enable identification of single individuals. Therefore, in order to approve the study, the Regional Ethical Review Board in Lund made considerable restrictions regarding access to the data. This means we will not be able to provide other researchers with our data. However, as our database is compiled by register data only, other researchers may contact Statistics Sweden and the Swedish National Board of Health and Welfare to get access to the different registers included, and thereby recreate the database.

## Abbreviations

ASD: Autism Spectrum Disorder
ATC: Anatomical Therapeutic Chemical
CI: Confidence Interval
GABA: Gamma-Aminobutyric Acid
ICD-10: International Statistical Classification of Diseases and Related Health Problems 10th Revision
ID: Intellectual Disability
MSP: Moderate/Severe/Profound
RR: Relative Risk
